# Distinctive Structure of the EphA3/Ephrin-A5 Complex Reveals a Dual Mode of Eph Receptor Interaction for Ephrin-A5

**DOI:** 10.1371/journal.pone.0127081

**Published:** 2015-05-20

**Authors:** Garry Jason Forse, Maria Loressa Uson, Fariborz Nasertorabi, Anand Kolatkar, Ilaria Lamberto, Elena Bianca Pasquale, Peter Kuhn

**Affiliations:** 1 Dornsife College of Letters, Arts and Sciences, University of Southern California, 3430 S. Vermont Ave., Suite 105 (110), MC3301, Los Angeles, CA, 90089–3301, United States of America; 2 Sanford-Burnham Medical Research Institute, 10901 North Torrey Pines Road, La Jolla, California, 92037, United States of America; 3 Department of Pathology, University of California San Diego, La Jolla, California, 92093, United States of America; University of Parma, ITALY

## Abstract

The Eph receptor tyrosine kinase/ephrin ligand system regulates a wide spectrum of physiological processes, while its dysregulation has been implicated in cancer progression. The human EphA3 receptor is widely upregulated in the tumor microenvironment and is highly expressed in some types of cancer cells. Furthermore, EphA3 is among the most highly mutated genes in lung cancer and it is also frequently mutated in other cancers. We report the structure of the ligand-binding domain of the EphA3 receptor in complex with its preferred ligand, ephrin-A5. The structure of the complex reveals a pronounced tilt of the ephrin-A5 ligand compared to its orientation when bound to the EphA2 and EphB2 receptors and similar to its orientation when bound to EphA4. This tilt brings an additional area of ephrin-A5 into contact with regions of EphA3 outside the ephrin-binding pocket thereby enlarging the size of the interface, which is consistent with the high binding affinity of ephrin-A5 for EphA3. This large variation in the tilt of ephrin-A5 bound to different Eph receptors has not been previously observed for other ephrins.

## Introduction

The Eph receptor tyrosine kinase family regulates a variety of physiological processes in the developing and adult organism, ranging from tissue patterning and angiogenesis to glucose homeostasis and bone remodeling, while its dysregulation has been implicated in diseases such as cancer[1,2]. One of the Eph receptors, EphA3, was recently found to be among the most frequently mutated genes in a study of 188 lung adenocarcinomas [3]. In addition, many EphA3 mutations have been found in a number of other cancers (www.cbioportal.org). EphA3 cancer somatic mutations are distributed throughout the domains of the receptor and some have been shown to impair ephrin ligand-dependent receptor signaling, suggesting a tumor suppressor role for the EphA3/ephrin interaction, which has been substantiated by functional studies [4–6]. In addition, EphA3 has been reported to be widely upregulated in the tumor stroma and in some types of cancer cells [7,8]. As EphA3 likely plays a role in cancer malignancy, there is considerable interest in understanding the mechanisms underlying EphA3 structure and function. This information will help elucidate the role of EphA3 in cancer cells, which is poorly understood, and design strategies to target EphA3 for cancer treatment.

The Eph receptors are divided into A and B classes, which have different binding preferences for the membrane-anchored ephrin ligands [9]. EphA and EphB receptors generally bind ephrin-A and ephrin-B ligands, respectively, although there are examples of inter-class binding [9]. The ligand-binding domain (LBD) of the Eph receptors comprises approximately 200 amino acids at the N-terminus of the extracellular region and is followed by a cysteine-rich region and two fibronectin III repeats [9]. The receptor-binding domain of the ephrins represents most of their extracellular region and is connected through a short linker segment to a GPI-anchor (ephrin-As) or a transmembrane segment (ephrin-Bs). Binding to ephrin ligands on opposing cells causes the Eph receptors to cluster, which involves only a subtle conformational rearrangement of the extracellular region and the formation of receptor-receptor interfaces [10,11]. Ephrin binding and clustering leads to activation of the Eph receptor intracellular kinase domain, leading to reciprocal phosphorylation of Eph receptor molecules in their cytoplasmic regions. The subsequent recruitment of SH2 domain-containing proteins and phosphorylation of cytoplasmic target proteins brings about a cascade of downstream cell signaling events [9].

The Eph receptors undergo conformational changes both in the ligand binding domain and the ATP binding pocket upon binding of ligands, leading to receptor activation. These two Eph receptor regions have been the focus of studies aimed at understanding the functional mechanism and for the purpose of drug development [12,13]. The ATP-binding pocket is highly conserved in all kinases, making it difficult to achieve high selectivity, despite the availability of structures for several Eph receptor kinase domains [14–17]. The extracellular ephrin-binding pockets of the Eph receptors can be targeted to either inhibit or activate the receptor [12,13]. Despite the high sequence conservation of the Eph receptor LBDs and the fact that each ephrin can bind multiple Eph receptors, individual receptors can be targeted specifically with peptides due to key differences in residues lining their ephrin-binding pockets [12,18]. We sought to elucidate the molecular determinants of the EphA3/ephrin binding interaction, which is the first step leading to ephrin-induced Eph receptor activation and signaling. We report the structure of the human EphA3 LBD in complex with its preferred ligand, ephrin-A5, which reveals a large interface with an extensive network of contacts.

## Results and Discussion

### Thermodynamic characterization of ephrin-A5-EphA3 binding

**Fig 1:**
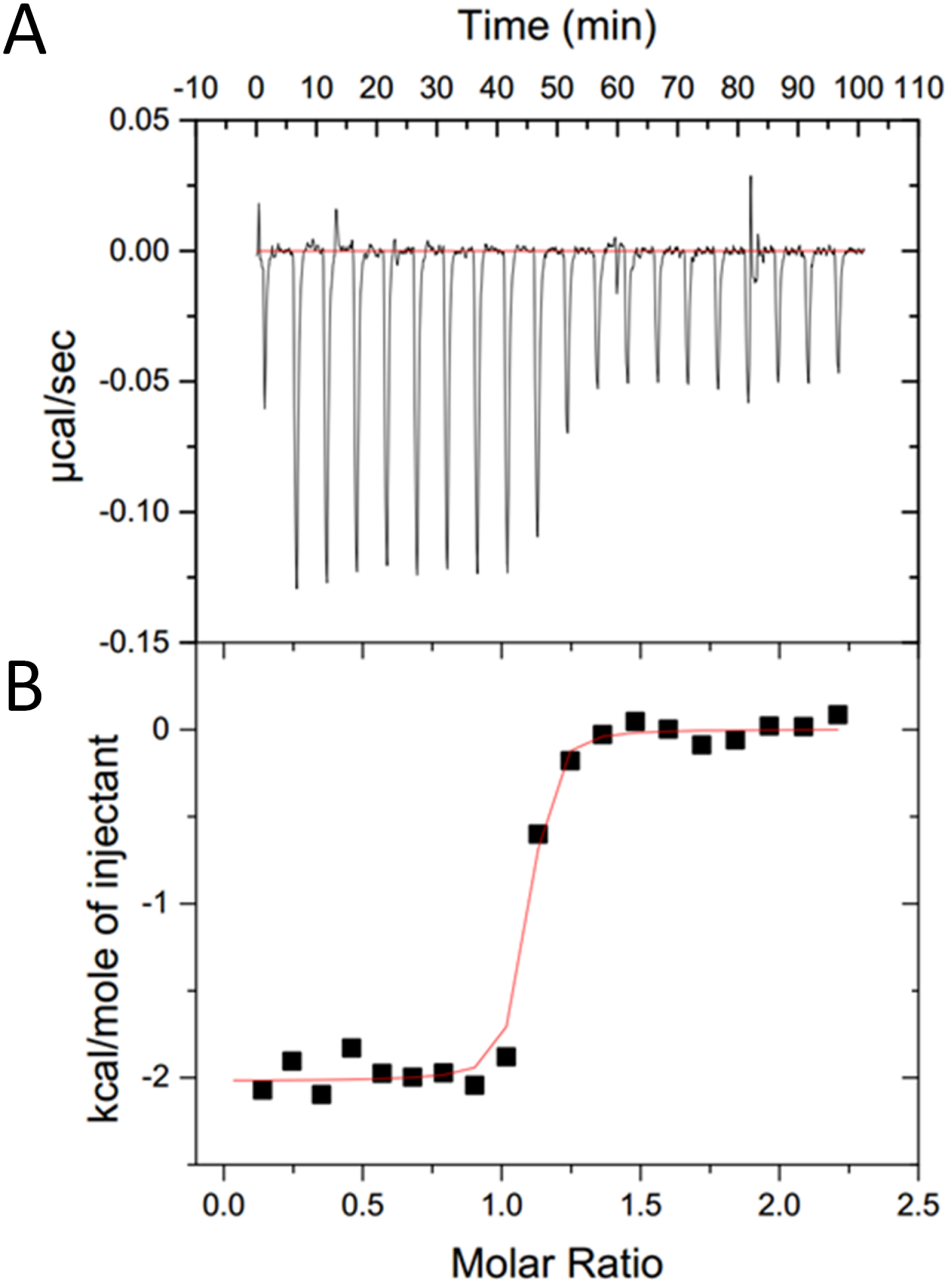
ITC analysis of ephrinA5-EphA3 binding. (A) Raw data showing the heat pulses resulting from a titration of ephrin-A5 (10 μM) in the calorimetric cell with an initial 5 μl injection of 100 μM EphA3 followed by 19 subsequent 15 μl injections. (B) Integrated heat pulses normalized per mole of injectant as a function of the molar ratio.

Isothermal titration calorimetry (ITC) experiments demonstrated that the EphA3 LBD binds ephrin-A5 in a process driven by both enthalpy and entropy (Table 1). Entropy is the primary driving force, likely due to the release of water when the hydrophobic ephrin-A5 GH loop docks into the hydrophobic ephrin-binding pocket of EphA3, thus increasing the entropy of the solvent, as previously observed for the EphB4/ephrin-B2 complex [19]. In contrast to the endothermic binding interaction observed for the EphB4/ephrin-B2 complex [19], however, EphA3 binds ephrin-A5 in an exothermic manner (Table 1). Thus, EphA3/ephrin-A5 binding benefits from both favorable entropic and favorable enthalpic contributions resulting in a low K_D_ of 9 ± 4 nM (Fig 1 and Table 1). This K_D_ value is similar to that previously reported for the entire EphA3 ectodomain using surface plasmon resonance [20]. The exothermic binding of ephrin-A5 to EphA3 observed by ITC is consistent with the extensive interface contacts observed in the crystal structure of the EphA3 LBD/ephrin-A5 complex.

**Table 1:** ITC data for EphA3-ephrin-A5 binding*.

ΔH	-2,145 ± 177 cal/mol
TΔS	8,855 ± 120 cal/mol
ΔG	-11,000 ± 297 cal/mol
K_D_	9 ± 4 nM

* Data are averages from 2 measurements ± SD.

### Overall structure of the EphA3/ephrin-A5 complex

**Fig 2:**
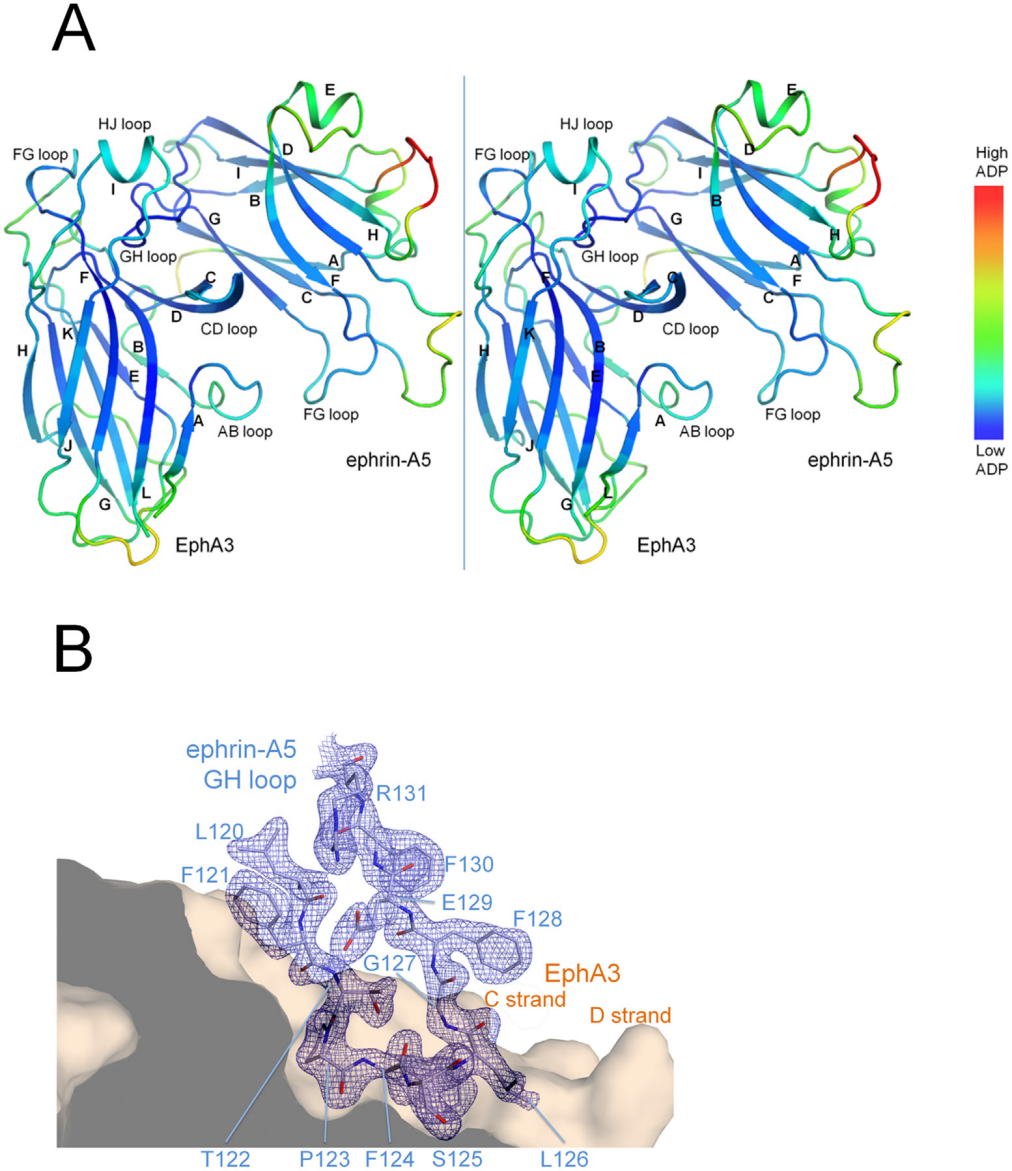
Crystal structure of the EphA3/ephrin-A5 complex. (A) Stereo image of the complex. The α-helices and β-strands are labeled for each protein, and residues are colored by atomic displacement parameters (ADPs). Loops along the interface are well ordered (low ADPs) compared to loops located at the periphery. (B) The electron density map for the ephrin-A5 GH loop is shown with a 2|F_obs_|-|F_calc_| map contoured at 1.5 σ as wire mesh for density within 2.1 Å from the center of the atoms. The ephrin-A5 GH loop is shown in the ephrin-binding pocket of EphA3 (depicted as a surface representation) in front the C^R^ and D^R^ strands (^R^ indicates the EphA3 receptor and ^L^ indicates the Ephrin A5 ligand). The HJ^R^ loop has been removed for clarity and would otherwise be situated above the ephrin-A5 GH loop. EphA3 residues have been removed at the sliced region depicted by a dark grey slab.

The human EphA3 LBD was crystallized in complex with the receptor-binding domain of the human ephrin-A5 ligand and the structure was determined to 2.26 Å resolution with an R_cryst_/R_free_ of 0.19/0.21, bond/angle residual mean square deviations (RMSD) of 0.004 Å/0.78°, and no Ramachandran outliers (Table 2). The crystals contained one heterodimer in the asymmetric unit with a well-ordered interface (Fig 2).

**Table 2:** Data collection and refinement statistics.

**Data collection**	
Space group	P4_3_ (*a* = *b* = 60.04 Å, *c* = 91.57 Å)
Resolution (Å)	40.00–2.26 (2.34–2.26)
*I/σI*	6.8 (2.0)
Completeness (%)	98.3 (93.5)
Redundancy	5.3 (4.0)
^a^ Rmerge(%)	19.0 (83.9)
**Refinement**	
Resolution	38.48–2.26
No reflections	15,062
^b^ *R*_work_(%) / *R*_free_ *(%)*	18.7 / 21.1
No. atoms	2737
Protein	2576
Ligand/ion	54
Water	107
R.m.s. deviations	
Bond lengths (Å)	0.004
Bond angles (°)	0.780
Average B value (Å^2^)	36.80
chain A	35.32
chain B	38.01
Waters	36.4
Ramachandran diagram (% in most favorable region)	97.0

Numbers in parentheses represent data in the highest resolution shell (2.34–2.26 Å).

^a^ R_merge_(*I*) = Σ_hkl_((Σ*i*|*I*_*hkl*,*i*_—[*I*_*hkl*_]|)/Σ_*i*_ *I*_*hkl*,*i*_).

^b^ R_cryst_ = Σ_*hkl*_|*F*_obs_|—|*F*_calc_|/Σ_*hkl*_|*F*_obs_|. *R*_free_ was computed identically, except that 5% of the reflections were omitted as a test set.

**Fig 3:**
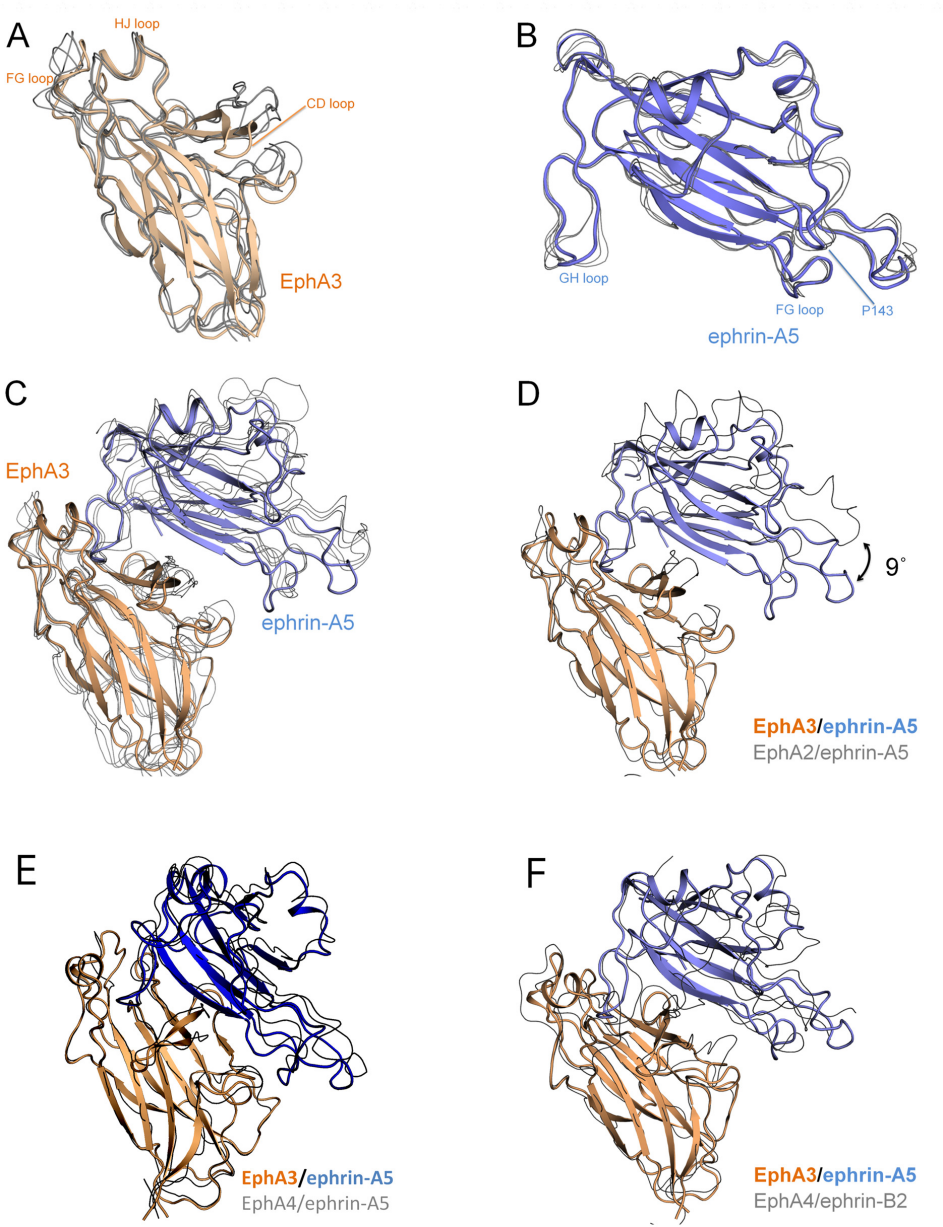
Ephrin-A5 is markedly tilted when bound to EphA3. (A) The ephrin-A5-bound EphA3 LBD (colored wheat) superimposes well to the EphA LBDs from other EphA/ephrin-A structures (depicted as grey lines; 0.5–0.7 Å RMSD to PDBIDs **3MX0** (EphA2/ephrin-A5), **3HEI** (EphA2/ephrin-A1), and **2WO3** (EphA4/ephrin-A2)). (B) Ephrin-A5 (colored light blue) superimposes well to previously available ephrin-A5 structures (0.5–0.6 Å RMSD to PDBIDs **1SHX** (unbound ephrin-A5), **1SHW** (EphB2/ephrin-A5), **3MX0** (EphA2/ephrin-A5), and **2X11** (EphA2/ephrin-A5)). (C) The EphA3/ephrin-A5 complex superimposes less well to other EphA/ephrin-A complexes (1.7–2.1 Å RMSD compared to **3MX0, 2X11, 2WO3** and **3HEI**). (D) Superposition of the EphA2/ephrin-A5 complex (PDBID **3MX0**) to just the EphA3 LBD reveals that ephrin-A5 is tilted by 9° in the EphA3/ephrin-A5 structure compared to its orientation in complex with EphA2. This is because the entire ephrin-A5 molecule, including the GH^L^ loop, is tilted. (E) The EphA3/ephrin-A5 complex is oriented similarly to an EphA4/ephrin-A5 complex (PDBID **4M4R**) that also shows a similar tilt of ephrin-A5 (Table 3). (F) The EphA3/ephrin-A5 complex is oriented similarly to the EphA4/ephrin-B2 complex (PDBID **2WO2**).

The individual EphA3 LBD and ephrin-A5 structures in our complex superimpose well to previously determined structures of other Eph receptor or ephrins, respectively (Fig 3A and 3B). This suggests a high degree of structural similarity, which is consistent with the high sequence conservation among family members. On the other hand, the EphA3/ephrin-A5 complex superimposes less well with a number of other Eph receptor/ephrin complexes (1.7–2.1 Å RMSD, Fig 3C).

**Table 3:** Ephrin tilt angles and interface areas in Eph/ephrin complexes.

PDB	Eph	Ephrin	Tilt angle	Resolution	Interface area (Å)	DE loop*	Reference
4L0P	EphA3	ephrin-A5	9°	2.3 Å	1,298	out	this article
3MX0	EphA2	ephrin-A5	0°	3.5 Å	847	in	[10]
***2X11***	***EphA2***	***ephrin-A5***	***5°***	***4*.*8 Å***	***992***	***in***	***[11]***
***4BKA***	***EphA4***	***ephrin-A5***	***9°***	***5*.*3 Å***	***1*,*167***	***in***	***[21]***
***4BK5***	EphA4**	ephrin-A5**	***0°***	***4*.*0 Å***	***810***	***in***	***[21]***
4M4R	EphA4	ephrin-A5	8°	3.1 Å	1,233	out	[22]
1SHW	EphB2	ephrin-A5	2°	2.2 Å	609	out	[23]
3HEI	EphA2	ephrin-A1	3°	2.0 Å	1,082	in	[24]
3MBW	EphA2	ephrin-A1	4°	2.8 Å	1,089	in	[10]
3CZU	EphA2	ephrin-A1	4°	2.6 Å	1,128	in	[10]
2WO3	EphA4	ephrin-A2	5°	2.3 Å	977	out	[25]
2WO2	EphA4	ephrin-B2	10°	2.4 Å	1,234	out	[25]
3GXU	EphA4	ephrin-B2	7°	2.5 Å	1,165	out	[26]
***4BKF***	***EphA4***	***ephrin-B3***	***3°***	***4*.*6 Å***	***1*,*221***	***out***	***[21]***
1KGY	EphB2	ephrin-B2	7°	2.7 Å	1,189	out	[27]
2HLE	EphB4	ephrin-B2	7°	2.0 Å	1,051	out	[19]

*Out, not part of the interface area; in, part of the interface area.

**Amine-methylated protein. Low resolution structures are marked with bold italic.

A notable difference is that ephrin-A5 is more tilted when bound to EphA3 compared to other EphA/ephrin-A complexes such as EphA2/ephrin-A5 (Fig 3D, Table 3). This mode of binding with a pronounced tilt has previously been observed only in structures of ephrin-A5 bound to EphA4 [21,22] (Fig 3E) or ephrin-B2 bound to Eph receptors [19,25–27] (Fig 3F and Table 3). Thus, EphA3 binds ephrin-A5 in a manner similar to EphA4 but different from EphA2 and EphB2 (Table 3). In previously determined structures of ephrin-A5 complexes with EphA2 or EphB2, the ephrin GH loop mediates the interaction with the Eph receptor LBD while separation from the receptor is maintained in the region below the GH loop. In contrast, the pronounced tilt of ephrin-A5 in our structure brings the ephrin ligand closer to the AB^R^ loop (^R^ indicates the EphA3 receptor and ^L^ indicates the Ephrin A5 ligand) and burying a larger surface area (as calculated with Protein Interfaces, Surfaces and Assemblies (PISA) [28] and Shape Correlation statistics (SC) [29] software). Indeed, ephrin-A5 bound to EphA2 buries a surface area of 847 Å^2^ (PDBID **3MX0**) [10], while it buries a larger surface area of 1,298 Å^2^ when bound to EphA3 (Table 3). Thus, the EphA3/ephrin-A5 structure shows that ephrin-A5 creates more contacts with EphA3 due to the larger interaction surface induced by the tilted binding mode.

In all high resolution Eph receptor/ephrin structures in which the ephrins are tilted, the DE loops of the Eph receptor are pushed away from the interface area (Table 3). This will reduce the interaction surface but generate closer and tighter contacts, which could potentially explain why some of the not-tilted ephrins show unexpectedly large interface areas. However, the tilt is not necessarily required for the shift of the DE loops, which is also seen in some of the Eph receptor/ephrin structures in which the ephrin is minimally tilted or not tilted.

**Fig 4:**
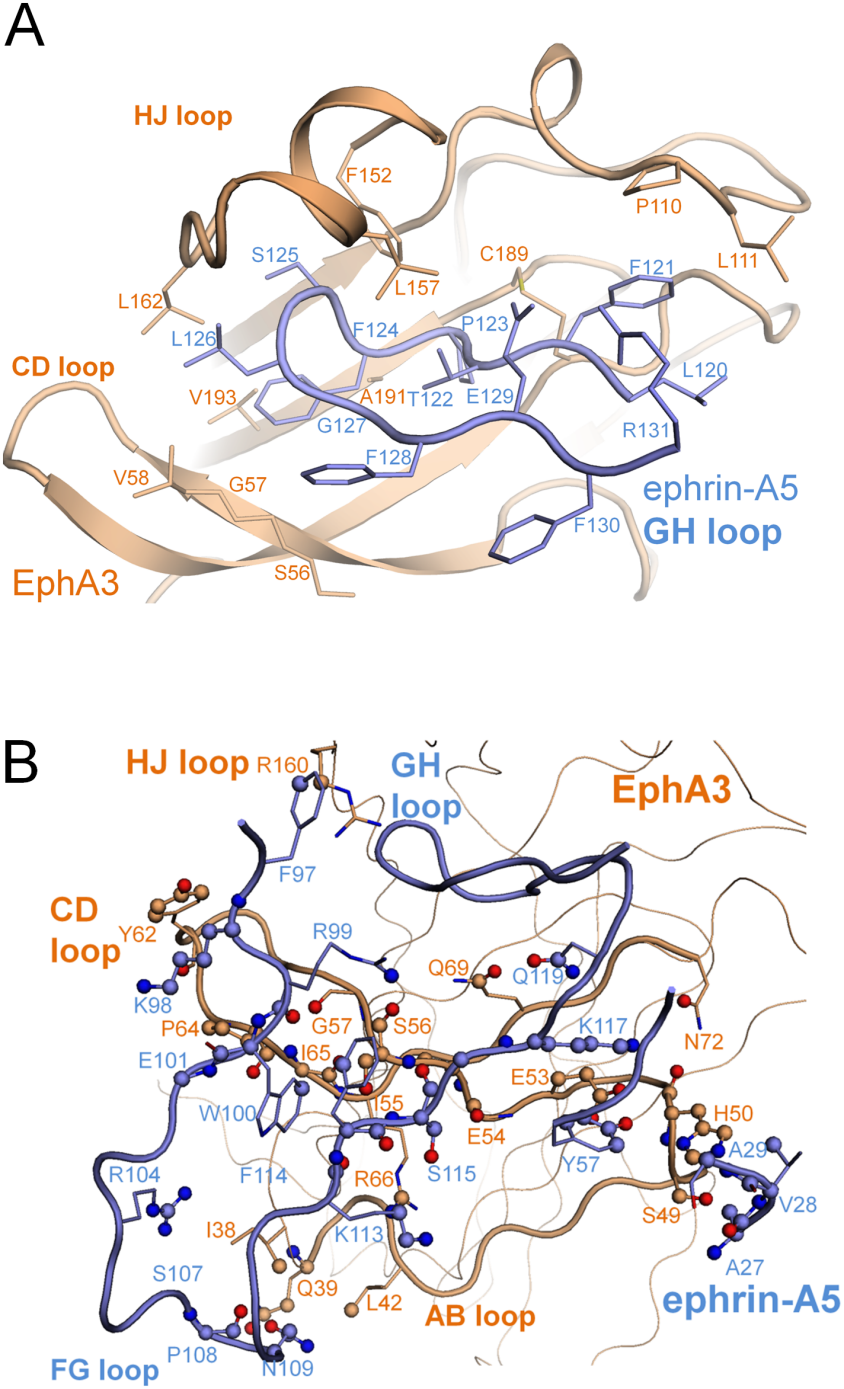
Features of the EphA3/ephrin-A5 interface. (A) The L111 cap. The ephrin-binding pocket of EphA3 (in wheat cartoon representation, with select residues represented by sticks) is extended at the top by residue L111^R^, which accommodates a shift in conformation of residue F121^L^. In the bottom part of the pocket, a sharp twist around residues S56^R^ and G57^R^ brings side chains from the C^R^ and D^R^ strands into contact with ephrin-A5. (B) The interaction between ephrin-A5 and EphA3 extends outside the ephrin-binding pocket. Atoms in contact with their binding partner are modeled as spheres, colored dark blue for N atoms or red for O atoms. Side chains of residues in the ephrin-GH loop (shown in Fig 4A) are hidden for clarity in Fig 4B.

The EphA3 C strand lining the bottom half of the ephrin-binding pocket is remarkably twisted, which enables extensive contacts with ephrin-A5 residues outside of the ephrin GH loop. The EphA3 C strand twists by 198°, primarily through the flexible residues S56 and G57 (Fig 4A). One consequence of this larger twist in EphA3 is that residues S49-V58 from the C strand are brought into contact with ephrin-A5 residues located below the ephrin-binding pocket (Fig 4B). The majority of these interactions are made by the EphA3 AB^R^ loop and the C^R^ and D^R^ strands with the ephrin FG loop and include 21 residues from ephrin-A5 contacting 26 residues of EphA3 (Table 4). This extensive network of interactions is consistent with the high enthalpy of EphA3/ephrin-A5 binding as determined by ITC (Table 1). Other Eph receptors that contain a similar Ser-Gly motif in this region, such as EphA7 (PDBID **3NRU**) and EphB receptors such as EphB2 (PDBID **1SHW**), have smaller twists (100°-150°) in their C strands. Solving the crystal structure of the unbound EphA3 LBD will be required to determine whether interaction with ephrin-A5 may be responsible for the unusual conformation of the EphA3 C strand.

**Table 4:** Ephrin-A5 interactions with EphA3.

Ephrin-A5^L^	EphA3^R^
**Residues not in the GH loopops**	
A27^SC^	H50^SC^
V28^SC^	H50^SC^
A29^BB^	H50^SC^
Y57^SC^	H50^SC^ **, H50** ^**SC**^, **E53** ^**SC**^
F97^SC^	R160^SC^
K98^SC^	Y62^SC^
W100^SC^	S56^SC^, P64^SC^
R104^SC^	I38^SC^
P108^BB^	I38^SC^
N109^BB^	I38^SC^, L42^SC^
F114^SC^	S56^SC^
S115^BB^	E54^BB^, I55^SC^, **S56** ^**SC**^, **S56** ^**BB**^
S115^SC^	E54^BB^
K117^SC^	**E53** ^**SC**^, I55^SC^
**Residues in the GH loop**	
*F121* ^*SC*^	*P110* ^*SC*^, *I 111* ^*SC*^
*T122* ^*SC*^	*L157* ^*SC*^
*T122* ^*BB*^	***R 104*** ^***SC***^, *F152* ^*SC*^
*P123* ^*SC*^	*Q69* ^*SC*^, *C189* ^*BB*^, *V190* ^*BB*^, *A191* ^*BB*^
*P123* ^*BB*^	***T102*** ^***SC***^, *T102* ^*BB*^, *F152* ^*BB*^, *A191* ^*SC*^
*F124* ^*SC*^	*G57* ^*BB*^, *V58* ^*BB*^, *Q69* ^*SC*^, *R 104* ^*SC*^, *F152* ^*SC*^, *V193* ^*SC*^
*S125* ^*SC*^	*F152* ^*SC*^, ***I161*** ^***BB***^, *L162* ^*SC*^, ***K163*** ^***BB***^
*S125* ^*BB*^	*T102* ^*SC*^, *L157* ^*SC*^
*L126* ^*SC*^	*R160* ^*BB*^
*L126* ^*BB*^	***R160*** ^***SC***^
*G127* ^*BB*^	***R160*** ^***SC***^
*F128* ^*SC*^	*R160* ^*SC*^
*E129* ^*SC*^	***R104*** ^***SC***^, *L157* ^*SC*^

BB, backbone; SC, side chain.

Modes of interaction are marked as follows: van der Waals interactions in plain text, hydrogen bonds in bold, and salt bridges in bold and underlined.

### Interaction of the ephrin-A5 GH loop with the ephrin-binding pocket of EphA3

**Fig 5:**
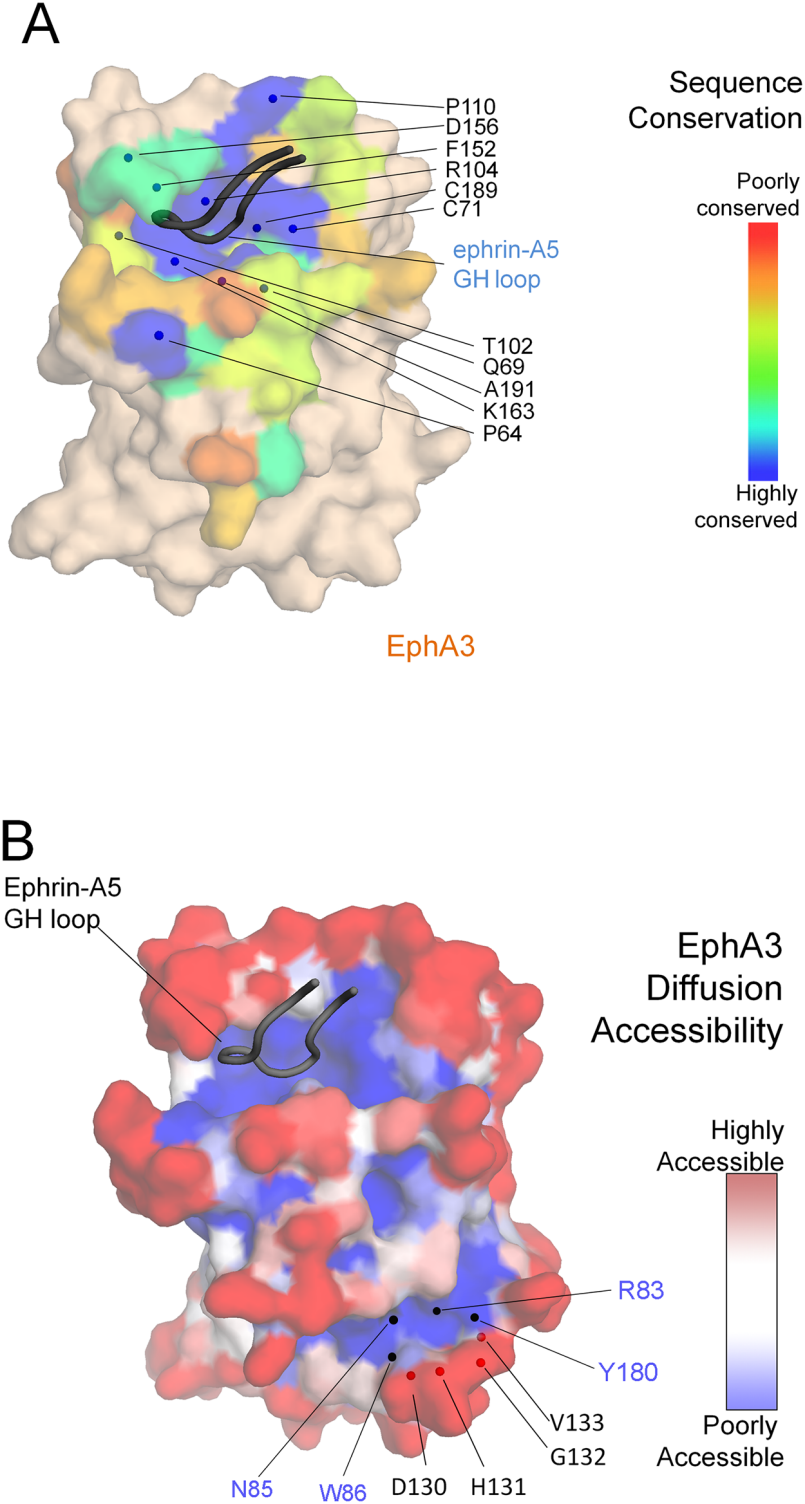
Surface properties of the EphA3 LBD. (A) Sequence conservation of the EphA3 LBD. Residues of EphA3 forming part of the interface with ephrin-A5 are colored by sequence conservation and other residues are colored wheat. The core of the EphA3 ephrin-binding pocket is lined by conserved residues, highlighted as blue spheres. EphA3 is shown in surface representation and the ephrin-A5 GH loop in cartoon representation. (B) Surface representation of the EphA3 LBD colored by diffusion accessibility. The two regions with poor diffusion accessibility (dark blue) are the ephrin-binding pocket (top left) and a channel near the previously described tetramerization surface [30] (bottom right). While the EphA3 LBD alone is not sufficient to form a heterotetramer, the structure nevertheless reveals a framework for residues D130, H131, G132 and V133, which have been proposed to be part of the tetramerization surface [30]. This framework includes residues R83, N85, W86 and Y180 located in a channel with poor diffusion accessibility.

Ephrin-A5 inserts its GH loop into the well conserved ephrin-binding pocket of EphA3 (Fig 5A). The receptor pocket forms a tight interface with the ephrin GH loop, with a significantly high surface complementarity score of 0.78 as measured by SC statistics [29]. The hydrophobic environment of the ephrin-binding pocket of EphA3 creates more direct contacts with the ephrin-A5 GH loop compared with other Eph receptors that have been crystallized in complex with less tilted ephrins, such as EphA2 and EphB2. Contacts are formed by EphA3 residues G57, V58, Q69, T102, R104, P110, L111, F152, L157, R160, I161, L162, K163, C189, V190, A191, V193 and ephrin-A5 residues F121 to E129 (Table 4). Nine residues of the ephrin GH loop penetrate the ephrin-binding pocket of EphA3 forming contacts with seventeen residues of the receptor, and six of the nine ephrin-A5 residues interact with EphA3 through salt bridges and hydrogen bonds (Table 4), consistent with tight binding. This comprises more than twice as many salt bridges and hydrogen bonds compared to other known EphA/ephrin-A5 structures without a tilt or with a small tilt.

As a consequence of the pronounced tilt of ephrin-A5, the FG loop of EphA3 tightens the ephrin binding pocket by pushing I109 of EphA3 towards ephrin-A5, forcing the side chain of F121^L^ to take a position oriented outward (Fig 4A). This places the side chain of F121^L^ under L111^R^, which caps the ephrin-binding pocket keeping the ephrin-A5 GH loop tight and stable inside the receptor. The strategic placement of L111^R^, which caps the ephrin-binding pocket, is unique because in all other Eph receptor crystal structures a proline occupies this position near the edge of the ephrin-binding pocket, leaving the pocket open.

### Additional features of the EphA3/ephrin-A5 interface

A previous EphA3 mutagenesis screen identified residues in the EphA3 LBD and cysteine-rich region that are important for tetramerization [30,31]. Without the cysteine-rich region, contacts from the LBD alone are not sufficient to drive tetramerization [30]. Indeed, as expected, our EphA3 LBD/ephrin-A5 complex forms a heterodimer as assessed by size exclusion chromatography (data not shown), consistent with previous studies [20]. Although the EphA3 LBD cannot drive tetramerization alone, structural analysis of previously identified residues in EphA3 involved in tetramerization can nevertheless be informative. Four of the five residues in the LBD that were previously found to be important for tetramerization (D130, His131, G132, and V133) lie across the edge of a channel containing residues (R83, N85, W86, and Y180) with a low diffusion accessibility (Fig 5B). This suggests that the channel may provide a framework important for positioning the residues of the tetramerizaiton surface and that the cancer mutations identified for some of these residues (cbioportal.org) may indirectly affect EphA3 tetramerization.

### The ephrin-A5-EphA3 complex

Ephrin-A5 adopts a distinct orientation upon binding EphA3, which is markedly more tilted compared to its orientation when bound to the EphB2 and EphA2 receptors. The tilt itself is not unprecedented, as ephrin-A5 adopts a similar tilt in some of the EphA4/ephrin-A5 complex structures [21,22] and ephrin-B2 also adopts large tilts in Eph receptor/ephrin-B2 complex structures [19,25–27]. However, ephrin-A5 is the first example of an ephrin that can bind to Eph receptors with a wide range of tilted angles. The markedly tilted binding mode of ephrin-A5 to EphA3 creates a larger and closer contact surface for binding. Much of that interface comes from residues exposed as a result of the tilt and the remarkably twisted C strand of EphA3, which forms substantial contacts with ephrin-A5 beyond the ephrin-A5 GH loop. The physiological relevance of the observed tilt is unknown, although as discussed above it is predicted to increase binding affinity. In addition, it may alter the orientation of the complex relative to the plasma membrane or other Eph/ephrin complexes. This may affect receptor oligomerization and downstream signaling.

## Experimental Procedures

### Production of human EphA3 in Escherichia coli

The gene for the EphA3 LBD comprising residues 29–200 was synthesized with codon usage optimized for *E*. *coli* and cloned into a modified pET32b vector (Novagen) by In-Fusion cloning (Clontech). Attempts to express EphA3 as a tag-free or thioredoxin-fused protein yielded protein that was mostly insoluble. The pET32b vector was modified to contain the fusion partner Glutathione S Transferase, a 10xHis tag, and a tobacco etch virus (TEV) protease cleavage site upstream, with the linker Gly-Ser-Gly inserted between each of these segments.

This plasmid was transformed into SHuffle T7 express cells (New England Biolabs). Attempts to express EphA3 in Origami cells (Novagen) or BL21 DE3 cells yielded insoluble protein. Four 2.8 L Fernbach flasks each containing 700 mL Terrific Broth (Research Products International) were inoculated at a ratio of 1:100 with an overnight culture grown from a single colony. The cells were grown to an OD_600 nm_ of 1.5, the temperature was lowered from 30°C to 18°C and protein expression was induced with 1 mM IPTG (Biopioneer). After 12 hr of expression, the cells were harvested by centrifugation at 5,000 g for 10 min at 4°C. All subsequent purification steps were carried out at 4°C.

The cells were resuspended in lysis buffer (50 mM Tris pH 8.0, 10 mM Imidazole, and 500 mM NaCl), protease inhibitor cocktail (Roche) and Benzonase (EMD Millipore). The cells were then lysed with a cell disruptor, centrifuged at 50,000 x g for 20 min, and the supernatant was filtered through a 0.2 μm filter. The filtered lysate was then incubated with 1.5 mL of settled Ni-NTA Superflow beads (Qiagen) on a rotator for 3 hr, packed into a column, washed with wash buffer (25 mM Tris pH 8.0, 30 mM Imidazole, 500 mM NaCl), and eluted with elution buffer (25 mM Tris pH 8.0, 250 mM Imidazole and 500 mM NaCl).

The Ni-NTA eluate was incubated with 3 mL of glutathione beads (Pierce) on a rotator overnight, washed with binding buffer and eluted with cleavage buffer (25 mM CHES pH 9.0, 0.01% Triton X-100, 150 mM NaCl, 10 mM reduced glutathione, and 1 mM oxidized glutathione). The protein in the glutathione eluate was concentrated to 5 mg/mL with an Amicon Ultra-15 10kDa (EMD Millipore) and cleaved with a protein:TEV protease ratio of 10:1 overnight. Triton X-100 greatly enhanced the solubility of the cleaved protein, while the low salt, high pH and reducing conditions in the cleavage buffer enhanced the efficiency of TEV cleavage. The EphA3 LBD protein was then purified by size exclusion chromatography (SEC) over a Superdex 75 16/60 column (GE) into SEC buffer (20 mM Tris pH 8.0, 150 mM NaCl). The yield was 7 mg of EphA3 per L of medium.

### Production of human ephrin-A5 in *Trichoplusia Ni* Tn5 cells

The gene for the ephrin-A5 receptor-binding domain comprising residues 26–166 was cloned from a Mammalian Gene Collection cDNA template (Thermo Scientific) into the pFastBac 1 vector (Life Technologies) by In-Fusion cloning. This vector was modified to contain a GP64 secretion signal, 10xHis tag, TEV cleavage site, and the Gly-Ser-Gly linker inserted between each of the segments.

The plasmid was transformed into DH10Bac cells (Life Technologies) to generate bacmid, and the bacmid was used to transfect Sf9 (*Spodoptera frugiperda*) cells with Cellfectin reagent (Life Technologies) to create a P0 baculovirus stock. From this, a P1 virus stock was generated with a titer of 10^9^ virus particles per mL and used to infect Tn5 cells at a multiplicity of infection of 5 in medium containing 2 x 10^6^ *Tn*5 cells per mL. Protein expression was carried out at 27°C for 48 hr. All subsequent purification steps were carried out at 4°C.

The culture was centrifuged at 1,000 g for 15 min, and the supernatant (medium) containing secreted protein was collected and mixed with protease inhibitor cocktail (Roche), 0.025% azide and 50 mL of 1 M Tris pH 8.0. This raised the pH of the medium to 7.5, and the medium was filtered through a 0.2 μm filter. The medium was then incubated with 1.5 mL Histrap Excel beads (GE) overnight, washed with lysis buffer, and eluted with elution buffer. The eluate containing ephrin-A5 was pooled, concentrated to 10 mg/mL with a Vivaspin Turbo 5 kDa Concentrator (Sartorius) and adjusted to 1 M urea from a 10 M stock. PNGase F was added to this sample at a molar ratio of 1:100 and incubated on a rotator for 1 hr at room temperature and then overnight at 10°C (the presence of 1 M urea increased the yield of deglycosylated ephrin-A5). The protein was then desalted into cleavage buffer using a HiTrap 26/10 desalting column (GE), concentrated, then cleaved and purified as described above for EphA3. An attempt to cleave ephrin-A5 without desalting into cleavage buffer yielded insoluble protein. The yield was 2 mg ephrin-A5 per L of medium.

### Complex formation and crystallization

The EphA3/ephrin-A5 complex was formed with a 1.5 molar excess of EphA3, and incubated on a rotator overnight at 4°C. The complex was loaded over a Superdex 75 16/60 column, and peak fractions belonging to the heterodimer were pooled and concentrated (Sartorius) to 10 mg/mL for crystallization.

Crystal trays were set up at 19°C by sitting drop vapor diffusion, and initial crystals were observed from the NeXtal AmSO4 Screen (Qiagen). These crystals were clusters of needles, and a fine screen was used to determine an optimal crystallization condition of 2.05 M (NH_4_)_2_SO_4_, 0.1 M Tris pH 8.0, and 5 mM CaCl_2_. This condition along with a lower protein concentration of 5 mg/mL resulted in larger single crystals of dimensions 8 x 8 x 100 μm in 400 nL drops. Crystals were cryo-preserved in liquid nitrogen with 25% glycerol as a cryoprotectant.

Crystals formed in space group P4_3_ with one subunit each of EphA3 and ephrin-A5 in the asymmetric unit. A dataset was collected at the Advanced Photon Source. Images were processed with HKL2000[32]. The phase problem was solved with PHASER [33] (CCP4i [34]), using the ephrin-A5 subunit from the structure of EphB2/ephrin-A5 (PDB ID **1SHW**) as the search model. The residues for EphA3 were built with Arp/wARP [35] (CCP4i), and the structure was improved by iterative rounds of model building with Coot [36] and refinement with Phenix [37]. Sequence conservation analysis was carried out with AL2CO [38]. Protein interfaces were analyzed for surface complementarity[29] and surface area buried [28], and structural alignments were calculated and figures were generated using PyMOL [39].

### Isothermal titration calorimetry

The EphA3 and ephrin-A5 proteins were dialyzed in 50 mM Tris-Cl, pH 7.8, 150 mM NaCl prior to use in two independent ITC experiments, both performed at 25°C. The first experiment was performed using a MicroCal VP-ITC instrument (Malvern Instrument, UK) according to the following protocol: 2 ml of 10 μM ephrin-A5 in the calorimetric cell was titrated with one injection of 5 μl EphA3 at a concentration of 100 μM followed by 19 additional injections with 15 μl volumes of EphA3. The second experiment was carried out with a MicroCal iTC200 instrument (Malvern Instrument, UK) according to the following protocol: 300 μl of 46 μM ephrin-A5 in the calorimetric cell was titrated with 19 injections of 2 μl EphA3 at a concentration of 460 μM. Microcal software was used to analyze the data from both experiments. ITC data were fitted using ‘one set of sites’ binding model.

### Three-atom tilt angle measurement

For the measurement of tilt angle reported in Table 3, Eph/Ephrin complexes were first trimmed to contain only the residues corresponding to those in the EphA3/ephrinA5 complex (4L0P). Each complex was then superimposed on EphA3 and the tilt of the ephrin was measured in relation to EphA3. Two EphA3 residues (F152^R^ and Y180^R^) that superimposed well with the corresponding residues in all other Eph receptors, and a residue from the interface area of ephrin-A5 (L112^L^) or the corresponding residue in other ephrins were selected for a standard three atom angle measurement. The angle between L112^L^ and Y180^R^ was measured by having residue F152^R^ as the mid point. Since the structure of EphA2/EphrinA5 (3MX0) showed the lowest degree of tilt, we considered chose the angle of ephrin-A5 in this complex as the 0° reference point. It should be noted that these measurements are associated with some uncertainty due to the different rotation and translation of molecules, loops and domains. Nevertheless, they provide a useful indication of the ephrin tilt angle in Eph receptor/ephrin complex structures.

### Accession number

Coordinates and structure factors for the EphA3 LBD/ephrin-A5 receptor-binding domain complex were deposited into the PDB with accession number ID 4L0P.
